# High Prevalence and New Genotype of *Coxiella burnetii* in Ticks Infesting Camels in Somalia

**DOI:** 10.3390/pathogens10060741

**Published:** 2021-06-12

**Authors:** Dimitrios Frangoulidis, Claudia Kahlhofer, Ahmed Shire Said, Abdinasir Yusuf Osman, Lidia Chitimia-Dobler, Yassir Adam Shuaib

**Affiliations:** 1 Bundeswehr Medical Service Headquarters VI-2, Medical Intelligence & Information, Dachauer Str. 128, 80637 Munich, Germany; DimitriosFrangoulidis@Bundeswehr.org; 2 Bundeswehr Institute of Microbiology, Neuherbergstr. 11, 80937 Munich, Germany; claudiakahlhofer@bundeswehr.org; 3 College of Veterinary Medicine, East Africa University, Bosaso P.O. Box 111, Somalia; walbaq1@gmail.com; 4 The Royal Veterinary College, University of London, Hawkshead Lane, North Mymms, Hatfield, Hertfordshire AL9 7TA, UK; aosman@rvc.ac.uk; 5 Department of Parasitology, Institute of Zoology, University of Hohenheim, Emil Wolff-Strasse 34, 70599 Stuttgart, Germany; 6 College of Veterinary Medicine, Sudan University of Science and Technology, P.O. Box 204 Hilat Kuku, Khartoum North 13321, Sudan

**Keywords:** prevalence, molecular typing, *Coxiella burnetii*, ticks, camels, Somalia, MLVA

## Abstract

*Coxiella burnetii* is the causative agent of Q fever. It can infect animals, humans, and birds, as well as ticks, and it has a worldwide geographical distribution. To better understand the epidemiology of *C. burnetii* in Somalia, ticks infesting camels were collected from five different regions, including Bari, Nugaal, Mudug, Sool, and Sanaag, between January and March 2018. Collected ticks were tested for *C. burnetii* and *Coxiella*-like endosymbiont DNA by using IS1111, *icd*, and *Com1*-target PCR assays. Moreover, sequencing of the 16S-rRNA was conducted. Molecular characterization and typing were done by *adaA*-gene analysis and plasmid-type identification. Further typing was carried out by 14-marker Multi-Locus Variable-Number Tandem Repeats (MLVA/VNTR) analysis. The investigated ticks (*n* = 237) were identified as *Hyalomma* spp. (*n* = 227, 95.8%), *Amblyomma* spp. (*n* = 8, 3.4%), and *Ripicephalus* spp. (*n* = 2, 0.8%), and 59.1% (140/237) of them were positive for *Coxiella* spp. While Sanger sequencing and plasmid-type identification revealed a *C. burnetii* that harbours the QpRS-plasmid, MLVA/VNTR genotyping showed a new genotype which was initially named D21. In conclusion, this is the first report of *C. burnetii* in ticks in Somalia. The findings denote the possibility that *C. burnetii* is endemic in Somalia. Further epidemiological studies investigating samples from humans, animals, and ticks within the context of “One Health” are warranted.

## 1. Introduction

*Coxiella burnetii* is an obligate intracellular zoonotic Gram-negative bacterium that causes Q fever [1,2,3,4,5]. It is considered one of the important emerging pathogens worldwide [6,7]. *C. burnetii* infection is sub-clinical in animals and does not result in any clinically detectable signs or symptoms except in pregnant animals where the infection has been associated with several reproductive disorders, including abortion, premature delivery, stillbirth, and weak offspring [6,8]. *C. burnetii* has been found in birth products (placenta and offspring), as well as in milk, vaginal mucus, faeces, and urine [6,9]. Moreover, it has also been found in blood and organs, such as the lungs, liver, and spleen [6]. The main reservoirs of *C. burnetii* are ruminants [6]. Its transmission to humans is through inhalation of contaminated aerosols, ingestion of raw milk, or contact with infected animals [6]. *C. burnetii* infection in humans is often asymptomatic/subclinical in up to 50% of cases or manifests as an acute febrile illness with pneumonia and/or hepatitis, depending on the geographical origin of the infection [10]. In a small proportion of patients (i.e., 1–2% of acute cases), the disease may progress to a chronic form that is clinically associated with patients having an endocarditis like illness [11]. Moreover, recent studies have also demonstrated the importance of vascular infection affecting the intima of large arteries [12,13,14]. The risk of *C. burnetii* infection is higher for people living in rural regions, animal owners/herders, veterinarians, and slaughterhouse/abattoir workers [6].

In ticks, *C. burnetii* has mostly been detected in *Amblyomma* spp., *Rhipicephalus* spp., *Ixodes* spp., and *Dermacentor* spp. [2,5]. Nevertheless, *C. burnetii* can naturally infect more than 40 different tick species [2,15]. These ticks can transmit the pathogen vertically (i.e., transstadial or transovarial) and horizontally, via bites or in faeces, to wild mammals and birds [2,15]. Infected ticks play a major role in the natural cycle of *C. burnetiid* infection and thus represent a principal vector and reservoir [2].

In Somalia, the ecology and epidemiology of *C. burnetii* are not well understood due to the scarcity and under representation of research studies exposing an already vulnerable population to a high burden of zoonotic diseases [16,17,18]. We therefore aimed to identify and screen hard tick samples collected from camel populations in five states of Somalia for *Coxiella* spp.

## 2. Results

A total of 237 ticks were collected from camels in five states in Somalia, including Mudug, Sanaag, Bari, Sool, and Nugaal. Nearly all identified ticks were *Hyalomma* spp. (*n* = 227, 95.8%). Only eight (3.4%) were identified as *Amblyomma* spp. and two (0.8%) as *Ripicephalus* spp.

All samples were screened for *C. burnetii* and *Coxiella*-like endosymbionts DNA using IS1111-PCR, and 59.1% (140/237) samples were positive, of which 138 (98.6%) were *Hyalomma* ticks and 2 (1.4%) were *Amblyomma* ticks. Using *icd*- and *com1*-PCR, still 19.0% (45/237) of the samples were positive for *Coxiella* spp. However, due to the extreme low amount of specific DNA in most of the samples (i.e., 71.4% showed PCR cycle threshold/Ct-values higher than 35), only 4 (2.9%) samples with a higher DNA amount were selected for further genomic classification.

Confirmation of *C. burnetii* with 16S rRNA-PCR was successful, and further classification based on the acute disease antigen A (*adaA*)-PCR revealed an *adaA*-negative genotype (all samples were *adaA*-negative). In addition, the screening for *C. burnetii* specific plasmid types showed that all samples harbour the QpRS-plasmid.

Enhanced genomic typing using 14-marker Multi-Locus Variant Analysis (MLVA) could amplify successfully 11 of the 14 MLVA markers (ms/microsatellites marker). Uploading the repeat pattern data to the CoxBase online platform (https://coxbase.q-gaps.de, accessed on 26 April 2021) revealed a probably new incomplete (i.e., not all markers gave an amplification result) genotype which was preliminarily named D21 (Table 1).

**Figure 1:**
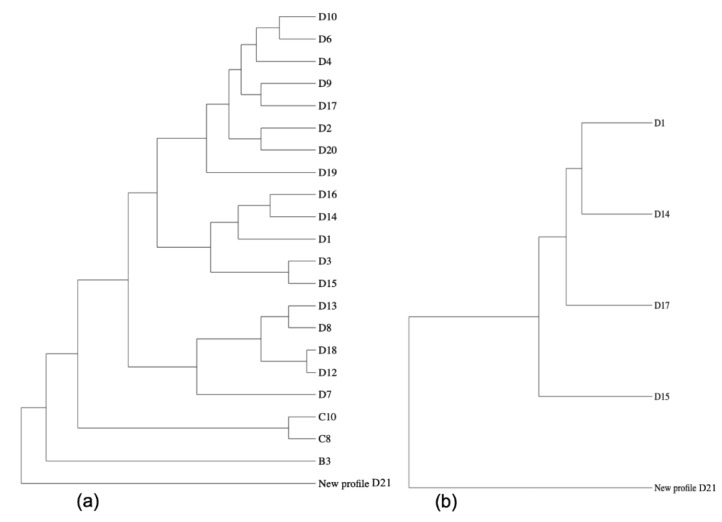
Phylogenetic comparison of the MLVA-pattern of the *C. burnetii* identified in this study when analysed via the CoxBase-online platform (https://coxbase.q-gaps.de, accessed on 26 April 2021) using PhyD3 (https://phyd3.bits.vib.be/, accessed on 26 April 2021). (**a**) The new genotype (proposed name D21) is an outgroup due to missing microsatellite (ms)-markers, when maximal distance is not limited. (**b**) Nearest neighbours displayed when maximal distance value is limited to five.

Comparison with the stored genotype information of other *C. burnetii* strains in the database showed a close relation between the newly identified genotype herein and a chronic strain genotyped from Saudi Arabia, with 9 out of 11 perfect matches, in addition to another chronic Q fever isolate from France, matching with 10 out of 11 microsatellites (ms) markers (Figure 1).

## 3. Discussion

*C. burnetii* infection, known as Q fever, has been reported in domestic and wild animals, birds, and in a wide variety of tick species [11,19]. Moreover, *C. burnetii* infection is globally a major public health problem with infections being reported sporadically, as outbreaks or endemic disease [6,19,20,21]. However, little is known about *C. burnetii* in many resource-limited countries and this is also true for Africa, where limited diagnostic resources are resulting in a significant underestimation of the disease [22], although some studies have clearly demonstrated the importance of Q fever [23,24]. Therefore, this study investigated tick samples collected from camels in five states of Somalia for *C. burnetii*.

By screening for the *C. burnetii* multicopy target, the insertion sequence IS1111, more than half of the tested tick samples were *Coxiella*-positive (140/237, 59.1%). This impressive molecular prevalence result, when using a sensitive Taqman-probe-assay, could not—as expected—be confirmed with PCR’s using single copy gene targets, such as *icd* and *com1*. More than 92% (129 from 140) of the samples showed a Cycle threshold (Ct) higher than 33, representing the low amount of *Coxiella* DNA in the collected tick samples, resulting in dropouts of the single copy gene target PCRs. Although ticks are not the major source of infection, neither for animals nor humans [19], this finding is of high importance and is very remarkable. It clearly shows the high burden and probably of the endemicity of *Coxiella* spp. in the study area. It further highlights the need for additional epidemiological studies within the context of “One Health” (i.e., investigate samples from humans, domestic and wild animals, birds, and ticks). This will provide a better insight into the ecology and distribution of this important zoonotic pathogen. A few former studies conducted in Somalia reported the prevalence of *C. burnetii*, using serological methods, with a positivity ranging from 25% to 37% in humans and up to 12.1% in sheep and goats [4,16,17,18], whereas this study is the first of its kind reporting direct evidence of the existence of the *C. burnetii* pathogen. In other parts of the world, the prevalence of *C. burnetii* in ticks varied from 1.6% to 5.5% [19,25,26,27,28]. Nevertheless, in a study from Kenya, five out of 10 pools of *Haemaphysalis leachi* ticks collected from domestic dogs were found to be positive for *C. burnetii* [29]. Furthermore, a study in rural Senegal identified up to 37% IS1111-positive *Amblyomma variegatum* ticks [30].

Results of the genotyping using MLVA/VNTR analysis showed a new genotype that has never been reported before. This new genotype is closely related to a *C. burnetii* strain isolated in Saudi Arabia from a patient with chronic Q fever and heart valve infection [31]. The new genotype is also related to genotypes from France and Russia [32,33]. On one hand, this finding confirms that MLVA/VNTR typing is a powerful tool that can offer a good understanding of the molecular epidemiology of *Coxiella* spp. However, it ideally requires high quality and quantity DNA with a PCR cycle threshold/Ct of less than 33; in addition, not all markers behave equally according to their sensitivity in identification of microsatellite repeats. Furthermore, there is another challenge related to MLVA/VNTR typing, which is how to deal with missing microsatellite-marker results, as this influences the correlation of different typing patterns. Especially in samples with a low DNA-amount, very often no differentiation between true negative ms-marker positions and missing typing PCR-signals is possible. Therefore, the measuring of genetic distances between strains/isolates should primarily be based on identified tandem repeat marker patterns (see [33]). The MLVA-analysis method used there, and in the here presented study, fits best with the algorithm for highly polymorphic tandem repeat loci developed by Shriver et al. [34].

In this study, the observed *C. burnetii*-positive ticks were *Hyalomma* spp. (*n* = 138) and *Amblyomma* spp. (*n* = 2). This confirms the findings of Deavaux et al. [4] and Bellabidi et al. [35], who indicated that *C. burnetii* was mostly detected in *Hyalomma* spp. infesting dromedary camels. Nevertheless, *C. burnetii* has been detected in many other hard and soft ticks collected from humans, cattle, sheep, and goats, as well as from wild animals, such as deer, and from birds [5,26,27,28,29,30,36]. These ticks included, for instance, *Ripicephalus* spp., *Amblyomma* spp., *Haemaphysalis* spp., *Ixodes* spp., *Dermacentor* spp., and *Ornithodoros* spp.

In conclusion, this is the first report of the occurrence of *C. burnetii* in ticks collected from domestic camels in Somalia. The findings of the study show that this query agent might be endemic in the study area. All *C. burnetii*-positive ticks were *Hyalomma* spp., except for two *Amblyomma* spp. Future studies should investigate ticks, samples of blood, and milk from animals (e.g., cattle, sheep, goats, and camels), and samples from humans as well. Furthermore, characterization of *C. burnetii* isolates from Somalia are warranted to obtain more insights related to the pathogen specific genomic properties and molecular epidemiology.

## 4. Materials and Methods

### 4.1. Study Area

**Figure 2:**
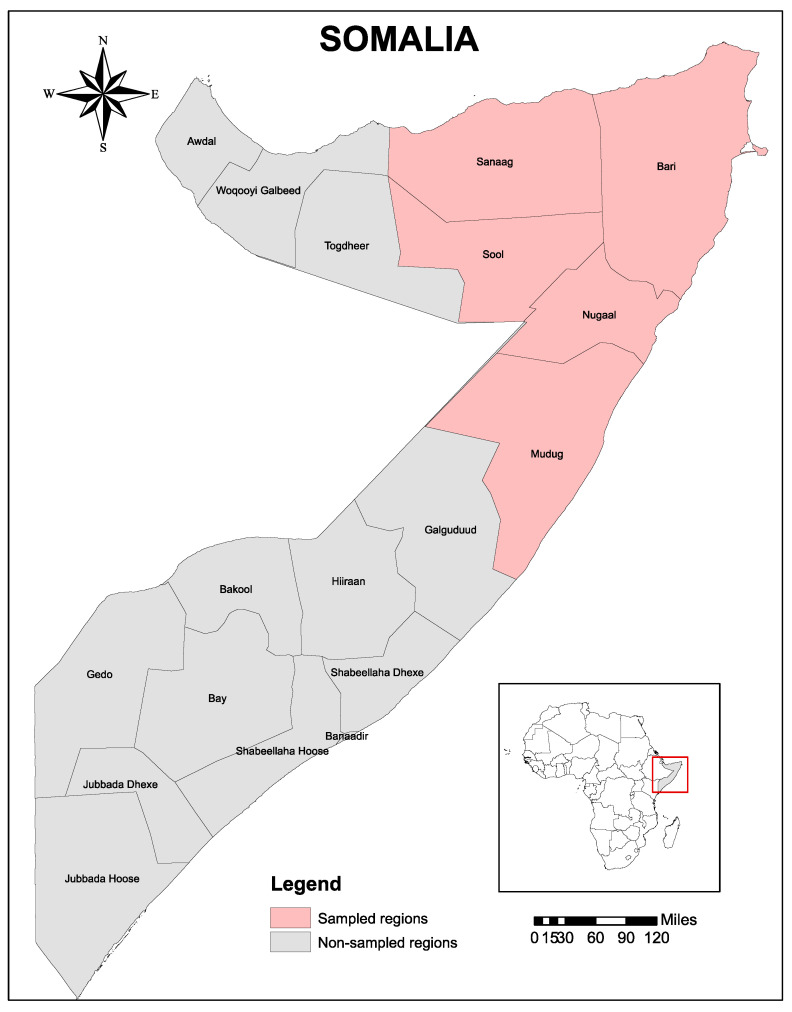
Map of the study area. The sampled five regions, including Bari, Nugaal Mudug, Sool, and Sanaag, of Somalia are indicated in pink. The map was created using ArcGIS v. 10 (esri Inc., Redlands, CA, USA).

This study was conducted in five states of Somalia that include Mudug, Sanaag, Bari, Sool, and Nugaal (Figure 2). Bari and Nugaal are under the administration of Puntland State, which is bordered by Somaliland State to its west, the Gulf of Aden in the north, the Guardafui Channel in the northeast, the Indian Ocean in the southeast, the Galmudug region in the south, and the Somali region of Ethiopia in the southwest [37,38]. The study area is characterized by alternating wet and dry seasons with predictable but erratically distributed rainfall during the wet season [37,38]. The average rainfall is neither sufficient nor reliable enough to produce staple crops. Conversely, the interaction of climatic and ecological factors could only support livestock production, which is predominant throughout Somalia and contributes significantly to the national economy. Livestock in the investigated five states of Somalia are indigenous breeds of goats, sheep, camels, and cattle that are more adaptable to arid environment. The livestock production system is exclusively nomadic pastoralism, and rangeland is communal or free grazing throughout the country. Livestock numbers are subject to many factors that include vicious cycles of major droughts and disease epidemics [37,38]. The total number of animals in Puntland is estimated to be nearly 18,000,000 heads, including 10.7% camels, 2.3% cattle, 35.4% sheep, and 51.6% goats. However, no census has been carried out since 1988, and the above data is derived from an estimated growth rate of 0.07% in camels, 0.01% in cattle, 0.012% in goats, and 0.021% in sheep. With its strategic location at the Horn of Africa, Puntland remains one of the principal main hubs for livestock exportation in Somalia and connects the Gulf states, South and Central Somalia, Ethiopia, Kenya, and Yemen [37,38].

All investigated animals were domesticated camels, and generally, in Somalia, camels are raised for milk and meat. They also indicate social status and wealth and are used in events such as marriage to pay dowry.

### 4.2. Study Design and Tick Samples Collection

This cross-sectional study was conducted between January and March 2018 in five states of Somalia, namely Bari, Nugaal, Mudug, Sool, and Sanaag. Tick samples were collected from camels of different age groups during routine veterinary and animal health care activities in the field.

Attached ticks were collected from camels using a pair of blunt forceps. Prior to collection, each animal was cast and restrained. Collected ticks from the same animal were kept separately in a universal bottle containing 70% ethanol, labelled indicating location, sex, and age [39,40].

### 4.3. Laboratory Procedures

#### 4.3.1. Tick Genera and Species Identification

Ticks were identified based on morphological characteristics according to Voltzit and Keirans [41], Apanaskevich and Horak [42,43,44], and Apanaskevich et al. [45].

Total nucleic acid was extracted using the MagNA Pure LC RNA/DNA Kit (Roche, Mannheim, Germany) in a MagNA Pure LC instrument (Roche), according to the manufacturer’s instructions. DNA was extracted from individual ticks or from pools containing 2 to 10 ticks per pool if ticks share the same developmental stage and species and had been collected from the same animal. The extracted total nucleic acid was stored at −80 °C until use.

When the morphology of a tick was not useful for identification, e.g., in the case of damage or if they were fully engorged ticks, the extracted DNA was used instead. The 16S rRNA gene was amplified using polymerase chain reaction (PCR) protocols and sequenced as described by Mangold et al. [46].

#### 4.3.2. Detection of *Coxiella burnetii*

The extracted total nucleic acid was further used to detect the presence of *C. burnetii* in ticks. Using different genomic targets, the samples were screened for *C. burnetii* and *Coxiella*-like endosymbionts [47,48]. After screening with IS1111-PCR, further *Coxiella* specific *icd* and *Com1*-target real-time PCR assays were done [36]. Positive results were confirmed with a species-specific 16S-rRNA PCR with sequencing and differentiation by performing a blast-analysis in the NCBI database [36]. Further genomic differentiation and typing were done with *adaA*-gene and plasmid-type identification [49].

#### 4.3.3. Molecular Characterization of *Coxiella burnetii*

Samples with enough DNA quality and quantity were genotyped with a 14-marker Multi-Locus Variable-Number of Tandem Repeats (MLVA/VNTR) analysis [26]. Identification and comparison of MLVA-genotype were done in the recent established online *Coxiella*-specific genotyping platform CoxBase (https://coxbase.q-gaps.de, accessed on 26 April 2021) [50]. This collection contains all of the known and published MLVA-genotypes of *C. burnetii*. The comparison is based on an algorithm for highly polymorphic tandem repeat loci developed by Shriver et al. [34]. Visualisation of phylogeny was done with PhyD3 (https://phyd3.bits.vib.be/, accessed on 26 April 2021), and to generate a breakdown of the strain relationship the maximal distance was limited to five. This means that only isolates of the CoxBase with up to five ms-loci having a different number of repeats to our MLVA-pattern were included in the analysis (excluding missing values).

## Figures and Tables

**Table 1:** Results of the CoxBase comparison of the newly identified MLVA-genotype pattern (first line of the table).

4	6	nr *	7	6	nr	nr	3	3	5	5.5	3	7	2	D21 (New)
ms01	ms03	ms01	ms20	ms22	ms23	ms24	ms26	ms27	ms28	ms30	ms31	ms33	ms34	Genotype
4.0	6.0	11.0	7.0	6.0	9.0	14.0	3.0	3.0	6.0	5.5	3.0	7.0	2.0	D14
4.0	6.0	11.0	7.0	6.0	−1.0	17.0	−1.0	3.0	8.0	5.5	3.0	7.0	2.0	D15
4.0	6.0	8.5	7.0	6.0	−1.0	10.0	3.0	4.0	5.0	5.5	3.0	6.0	2.0	D17
4.0	6.0	11.0	7.0	6.0	−1.0	0.0	−1.0	3.0	7.0	5.5	3.0	6.0	2.0	D8
4.0	6.0	13.0	7.0	6.0	9.0	8.0	3.0	3.0	6.0	5.5	3.0	6.0	2.0	D1
3.0	6.0	10.5	6.0	6.0	9.0	29.0	3.0	3.0	5.0	5.5	3.0	5.0	2.0	B3
4.0	6.0	11.0	7.0	6.0	9.0	14.0	3.0	3.0	6.0	5.5	3.0	7.0	2.0	D14
4.0	6.0	8.5	7.0	7.0	−1.0	11.0	2.0	4.0	5.0	5.5	3.0	8.0	2.0	D10
3.0	6.0	11.0	7.0	6.0	9.0	3.0	−1.0	3.0	7.0	5.5	3.0	7.0	2.0	D12
4.0	6.0	11.0	7.0	6.0	9.0	0.0	−1.0	3.0	7.0	5.5	3.0	5.0	2.0	D13
4.0	6.0	13.0	7.0	6.0	9.0	15.0	4.0	3.0	6.0	5.5	3.0	6.0	2.0	D16
4.0	6.0	11.0	7.0	6.0	9.0	17.0	−1.0	3.0	8.0	5.5	3.0	6.0	2.0	D3
3.0	6.0	8.5	6.0	6.0	9.0	11.0	3.0	5.0	5.0	5.5	3.0	6.0	2.0	D9
4.0	6.0	8.5	6.0	7.0	4.0	11.0	2.0	4.0	5.0	5.5	3.0	7.0	2.0	D6
4.0	6.0	8.5	6.0	7.0	7.0	8.0	2.0	4.0	5.0	5.5	3.0	7.0	2.0	D4
4.0	6.0	9.0	6.0	6.0	7.0	11.0	2.0	3.0	4.0	5.5	4.0	7.0	2.0	D20
4.0	6.0	9.0	6.0	6.0	2.0	10.0	2.0	3.0	4.0	5.5	4.0	7.0	2.0	D2
3.0	6.0	11.0	7.0	6.0	9.0	3.0	−1.0	3.0	7.0	5.5	3.0	6.0	2.0	D18
4.0	6.0	9.0	6.0	6.0	10.0	11.0	2.0	3.0	4.0	5.5	4.0	3.0	2.0	D19
3.0	6.0	8.5	7.0	7.0	9.0	0.0	2.0	4.0	5.0	5.5	3.0	8.0	2.0	D7
4.0	6.0	9.0	6.0	6.0	5.0	15.0	4.0	2.0	7.0	5.5	3.0	9.0	8.0	C8
3.0	6.0	9.0	6.0	6.0	5.0	15.0	3.0	2.0	7.0	5.5	3.0	9.0	9.0	C10

* nr = no result. “−1” values = no repeat numbers identified. ms = microsatellites.

## Data Availability

The data presented in this study are available on request from the corresponding author.
